# Interleukin-1 Receptor Antagonist Protects Newborn Mice Against Pulmonary Hypertension

**DOI:** 10.3389/fimmu.2019.01480

**Published:** 2019-07-11

**Authors:** Christine B. Bui, Magdalena Kolodziej, Emma Lamanna, Kirstin Elgass, Arvind Sehgal, Ina Rudloff, Daryl O. Schwenke, Hirotsugu Tsuchimochi, Maurice A. G. M. Kroon, Steven X. Cho, Anton Maksimenko, Marian Cholewa, Philip J. Berger, Morag J. Young, Jane E. Bourke, James T. Pearson, Marcel F. Nold, Claudia A. Nold-Petry

**Affiliations:** ^1^Ritchie Centre, Hudson Institute of Medical Research, Clayton, VIC, Australia; ^2^Department of Paediatrics, Monash University, Clayton, VIC, Australia; ^3^Faculty of Medicine, University of Rzeszow, Rzeszow, Poland; ^4^Department of Pharmacology, Biomedicine Discovery Institute, Monash University, Clayton, VIC, Australia; ^5^Monash Micro Imaging, Hudson Institute of Medical Research, Clayton, VIC, Australia; ^6^Monash Newborn, Monash Children's Hospital, Melbourne, VIC, Australia; ^7^Department of Physiology–Heart Otago, School of Biomedical Sciences, University of Otago, Dunedin, New Zealand; ^8^Cardiac Physiology, National Cerebral and Cardiovascular Center Research Institute, Suita, Japan; ^9^Department of Pharmacy, Amsterdam UMC, Amsterdam, Netherlands; ^10^Imaging and Medical Beamline, Australian Synchrotron, Clayton, VIC, Australia; ^11^Centre for Innovation and Transfer of Natural Sciences and Engineering Knowledge, University of Rzeszow, Rzeszow, Poland; ^12^Centre for Endocrinology and Metabolism, Hudson Institute of Medical Research, Clayton, VIC, Australia; ^13^Department of Physiology, Biomedicine Discovery Institute, Monash University, Clayton, VIC, Australia

**Keywords:** pulmonary hypertension, bronchopulmonary dysplasia, interleukin-1 receptor antagonist, pulmonary vascular resistance, neonatal immunity, anti-inflammatory therapy, interventional immunology, preterm infants

## Abstract

Pulmonary hypertension secondary to bronchopulmonary dysplasia (BPD-PH) represents a major complication of BPD in extremely preterm infants for which there are currently no safe and effective interventions. The abundance of interleukin-1 (IL-1) is strongly correlated with the severity and long-term outcome of BPD infants and we have previously shown that IL-1 receptor antagonist (IL-1Ra) protects against murine BPD; therefore, we hypothesized that IL-1Ra may also be effective against BPD-PH. We employed daily injections of IL-1Ra in a murine model in which BPD/BPD-PH was induced by antenatal LPS and postnatal hyperoxia of 65% O_2_. Pups reared in hyperoxia for 28 days exhibited a BPD-PH-like disease accompanied by significant changes in pulmonary vascular morphology: micro-CT revealed an 84% reduction in small vessels (4–5 μm diameter) compared to room air controls; this change was prevented by IL-1Ra. Pulmonary vascular resistance, assessed at day 28 of life by echocardiography using the inversely-related surrogate marker time-to-peak-velocity/right ventricular ejection time (TPV/RVET), increased in hyperoxic mice (0.27 compared to 0.32 in air controls), and fell significantly with daily IL-1Ra treatment (0.31). Importantly, *in vivo* cine-angiography revealed that this protection afforded by IL-1Ra treatment for 28 days is maintained at day 60 of life. Despite an increased abundance of mediators of pulmonary angiogenesis in day 5 lung lysates, namely vascular endothelial growth factor (VEGF) and endothelin-1 (ET-1), no difference was detected in *ex vivo* pulmonary vascular reactivity between air and hyperoxia mice as measured in precision cut lung slices, or by immunohistochemistry in alpha-smooth muscle actin (α-SMA) and endothelin receptor type-A (ET_A_) at day 28. Further, on day 28 of life we observed cardiac fibrosis by Sirius Red staining, which was accompanied by an increase in mRNA expression of galectin-3 and CCL2 (chemokine (C-C motif) ligand 2) in whole hearts of hyperoxic pups, which improved with IL-1Ra. In summary, our findings suggest that daily administration of the anti-inflammatory IL-1Ra prevents the increase in pulmonary vascular resistance and the pulmonary dysangiogenesis of murine BPD-PH, thus pointing to IL-1Ra as a promising candidate for the treatment of both BPD and BPD-PH.

## Introduction

Improved survival of preterm infants with gestational ages as low as 23 weeks exposes ever more preterm infants to bronchopulmonary dysplasia (BPD). BPD is a severe inflammatory lung disease that affects up to 15,000 preterm infants each year in the USA (1, 2), with the highest incidence of 35–68% in the 0.5–1 kg birth weight group (3, 4). The diminished lung function of the preterm infant calls for intensive care with mechanical ventilation and oxygen supplementation, which each contribute to the development of the multifactorial pathophysiology of BPD (5) and to health complications that persist into adulthood (6, 7).

Pulmonary hypertension secondary to BPD (BPD-PH) can be considered the gravest complication of BPD as it substantially worsens the prognosis of afflicted infants. BPD-PH can eventually over-tax the right ventricle and limit cardiac output. Occurring in 15–30% of BPD patients (8), the deleterious consequences of BPD-PH are far worse than those of systemic hypertension, with a survival of just 50% 2 years after diagnosis in the severe cases (9).

The risk factors for PH, which overlap with those for BPD, include low gestational age, fetal growth restriction, oligohydramnios, prolonged mechanical ventilation, and oxygen dependency (9, 10). In normal lung development, alveolarization, and vascularization of the distal lung saccules run in parallel (11). BPD disrupts development by reducing the number of alveoli, and therefore the overall volume of lung tissue. In addition, it also causes a significant dysangiogenesis that markedly reduces the cross-sectional area of the pulmonary vascular bed and thereby increases pulmonary arterial blood pressure (9, 12, 13). The ongoing afterload of the pulmonary circulation eventually causes hypertrophy and remodeling of the right ventricle as well as of the walls of the pulmonary vessels, and subsequently a narrowing of the vascular lumen that further limits blood flow (14–16).

Strong evidence implicates inflammation as a key player in BPD and BPD-PH (17–19). Mediators such as VEGF and endothelin-1 are increased and lead to the development of immature and leaky capillaries (20, 21). Recognizing that BPD and BPD-PH are inflammatory diseases, in 2006 the American Academy of Pediatrics issued an urgent call for new anti-inflammatory BPD therapies (22), but with no safe and effective therapy forthcoming, in 2014 the call was renewed (23).

Our research has focused on inhibition of IL-1 as a potential therapy. We previously showed that the endogenous inhibitor of IL-1, interleukin-1 receptor antagonist (IL-1Ra) (24), ameliorates murine BPD induced by perinatal inflammation and hyperoxia (19, 25, 26). To advance the prospects for an anti-inflammatory treatment for pulmonary injury and cardiovascular complications, we extended our earlier study by examining whether the benefit IL-1Ra provides against BPD extends to the pulmonary vasculature and thereby also improves pulmonary vascular resistance, cardiac inflammation and fibrosis. In this study, we confirm that IL-1Ra not only improves alveolar structure (19) but also lung vascularization, thus ameliorating BPD-PH. Hence, IL-1 is a promising target for therapeutic intervention in inflammatory neonatal diseases such as BPD and BPD-PH.

## Materials and Methods

### Murine Model of BPD-PH

We used a previously published two-hit model to induce BPD/BPD-PH in mice pups (19, 26). Briefly, on day 14 of gestation (embryonic day 14; E14) pregnant C57BL/6J mice were given an intra-peritoneal (i.p.) injection of 150 μg/kg of lipopolysaccharide (LPS) to mimic maternal systemic inflammation (e.g., chorioamnionitis). Pups delivered naturally at term (G19-21) when their lungs are at the saccular stage of lung development (27), which is at an equivalent stage to the lungs of extremely preterm human infants (23–29 weeks' gestational age) who are at most risk of developing BPD (28). Our intention was to study the impact of BPD-PH during the alveolar stage of lung development, which spans postnatal day 5 to day 28 (d5-d28) in the mouse, similar to 32 weeks' gestation to 2–3 years of age in the human. Thus, we chose day 28 as the first time-point to assess the pulmonary vasculature and PH in our mice. To assess long-term outcomes, we chose a second time-point of day 60 in mice, which represents ~20 years in human age (29).

Within 24 h after birth, pups and dams were randomized into treatment groups of daily subcutaneous (s.c.) injections for 28 days of 10 mg/kg IL-1Ra or s.c. injections with an equal volume of saline (vehicle) and then exposed to either gas with a FiO_2_ of 0.21 (room air) or 0.65 (hyperoxia) for 28 d, for a total of four experimental groups: air saline, air IL-1Ra, hyperoxia saline, and hyperoxia IL-1Ra. Dams had unlimited access to food and water and were rotated between room air and hyperoxia groups in a 3 day cycle, to reduce effects from exposure to hyperoxia. In the long-term study group, daily treatment with IL-1Ra ceased at day 28 and animals of all groups were left in room air from day 28 until day 60 of life. Experiments for all timepoints were performed at a temperature of 22°C and humidity of 50–60% and light was cycled in a 12 h day/night rhythm. At day 5 pups were humanely euthanized by decapitation and at day 28 or day 60 by cervical dislocation.

### Murine Echocardiography

At 28 d, mice were anesthetized with isoflurane (3% isoflurane mixed with 0.5 L/min 100% O_2_ to induce anesthesia and then 1–1.5% isoflurane mixed with 0.5 L/min 100% O_2_ to maintain anesthesia). All echocardiography evaluations were performed by a single operator using the Vivid 7 advantage cardiovascular ultrasound system (GE Medical Systems, Milwaukee, WI, USA). 2-D guide M-mode echocardiographic examination of the left ventricle was performed using a 13-MHz linear transducer (i13L probe; General Electric Co) at a sweep speed of 100 mm/s. The mouse was placed on a heated pad and time of anesthesia was <10 min for all animals (30). Time-to-peak-velocity (TPV) expressed relative to right ventricular ejection time (RVET) (TPV/RVET) was calculated as an index of right ventricular (RV) function and pulmonary artery pressure, to which it is inversely correlated. Left ventricular (LV) function was determined by measuring internal diameters at end-diastole and systole (LVIDd, LVIDs) and fractional shortening.

### X-Ray Micro-Computed Tomography, Image Acquisition, and Analysis

After cervical dislocation, 28 day lungs were intubated via the trachea and the lungs were fixed with 4% PFA (pH 7.4, instilled at a pressure of 20 cmH_2_O). The lung was then removed from the thorax, kept in 4% PFA for a minimum of 24 h, and then stored in 70% ethanol. For *ex vivo* X-ray micro computed tomography (thereafter referred to as CT) the right superior lobe was stained in Lugol's iodine solution (31, 32) for a minimum of 24 h and then washed in 70% ethanol and embedded in agarose for imaging.

CT scans were conducted with the X-ray photon energy tuned to 35 keV using the “Ruby” detector in the 3B enclosure of the Imaging and Medical beamline (IMBL) at the Australian Synchrotron, which was designed and fabricated by the Laboratory for Dynamic Imaging, Division of Biological Engineering, Monash University. During the experiment, the system was tuned to produce 2,560 × 2,160 pixel images giving a field of view of 15 × 12 mm with 6.0 μm pixel size with the measured resolution of 20.1 μm. CT data acquisition for the samples consisted of 1,800 projections over a 180° axis. The scans included 40 images of each background (no sample in the beam) and dark-field (beam is off) contrasts both before and after the sample acquisition. The exposures were 180 ms per projection, and accumulated time taken to scan a single sample was ~6 min. Each image projection was then processed by subtracting electronic noise and background (sample—dark field/background—dark field) using the median of all 40 frames of the background and dark-field images.

Reconstruction of a vertical series of CT cross-section slices of the samples was then performed utilizing a Filtered Back-Projection algorithm on the processed images with phase retrieval and ring artifact suppression filter as necessary. Processing was performed on the MASSIVE high performance cluster (33) using XLI software (34). Reconstructed slices were stored as 32-bit float-point TIFF images and further converted into 8-bit integer volumes for 3D rendering and analysis. Images were rendered using Drishti (35). The software tools used in the pipeline include several open-source projects: ImageMagick (36) for noise removal, cropping and image format conversion, and CTas (37) for the background and dark-field removal.

Image stacks were saved as 8-bit 3D.tif image stacks and displayed as 3D volumes in Imaris (Bitplane AG) for data quality checks. The image stacks then underwent several pre-processing steps prior to the final branching analysis. Pre-processing of the images was necessary to fill the larger diameter blood vessels so that the Imaris Filament Tracer can detect the vessel inside rather than the vessel membranes. Edge detection of large vessels was performed in Fiji (38) by running two subsequent difference-of-Gaussian (DoG) algorithms with increasing diameter (*r*_1_ = 2, *r*_2_ = 5), followed by thresholding, binarizing, and despeckling on each plane of the image stack. Subsequently, Matlab (MathWorks Inc.) was used to fill large vessels by further despeckling, skeletonizing, removing skeleton fragments, then detecting the tips of the skeleton, and closing with nearest neighbor algorithm and filling all fully-enclosed areas. The original and the filled image stacks was then loaded in Imaris as two channels, a smoothed surface was created around the filled data set and used to remove potential artifacts of the filling algorithm in the red channel. In a final step, Imaris Filament Tracer was used to detect large vessels (“dendrites”) in the filled channel, followed by the detection of small vessels (“spines”) in the original image stack (green channel). Overlaying the original volume with the analyzed filament network provided a visual quality check of the branching analysis.

For statistical visualization and analysis of the obtained filament data, multiple data processing steps were performed to ensure comparability of individual lung data sets as well as comparability between different treatment groups. Firstly, for each lung, all detected vessels were grouped into defined ranges of vessel diameters. Detected vessels with <4 μm diameter were discarded from subsequent analysis due to resolution limitations of the applied imaging technique. Absolute number of vessels were binned by diameter size and expressed as percentage of the total number of vessels per lung lobe. To take into account differences between the treatments on vessel number, each group was then normalized to the mean of the total vessel count of the air vehicle group.

### Precision Cut Lung Slices (PCLS)

PCLS were prepared from 28 day old mice as previously described, with minor modifications (39). Immediately after euthanasia, mice were dissected to expose the heart and trachea. The right ventricle of the heart was injected with warmed gelatin (~1 ml, 8% in 1X HBSS/HEPES). The trachea was cannulated with a catheter containing two ports (24G Intima; Becton Dickinson, North Ryde, NSW, Australia). The lungs were then inflated with warmed agarose (~1.2 ml, 2% in 1X HBSS/HEPES), followed by a bolus of air (~0.4 ml). The agarose was solidified in 1X HBSS/HEPES at 4°C for 15 min, and the left lobe isolated and mounted on a vibratome (Compresstome; Precisionary Instruments, Greenville, NC). PCLS (150 μm thickness) were cut and maintained overnight in DMEM containing 1% penicillin–streptomycin solution (37°C, 5% CO_2_). PCLS were transferred to 1X HBSS/HEPES and mounted in customized chambers (~100 μl) where an artery was selected (~120 μm diameter). Using a gravity-fed system, PCLS were perfused with U46619 (3–1000 nM) or ET-1 (1–100 nM). Arteries were visualized under phase–contrast microscopy (Nikon Eclipse Ti-U [Nikon Instruments Inc., Melville, NY]; Pulnix CCD camera model TM-62EX [Jai, Miyazaki, Japan]). Changes in artery lumen area were captured in the form of digital images (744 × 572 pixels) recorded in time lapse (0.5 Hz) using image acquisition and analysis software (Video Savant; IO Industries, Inc., London, ON, Canada). Images were converted to TIFF files and analyzed in ImageJ using a grayscale threshold to distinguish between the artery lumen and surrounding tissue, with lumen area in each image calculated by pixel summation.

### Lung and Heart Preparation, Histology, and Immunohistochemistry (IHC)

After cervical dislocation, 28 day old mice were intubated via the trachea and the lung was inflated and fixed with 4% paraformaldehyde (PFA; pH 7.4, instilled at 20 cmH_2_O pressure). Thereafter both the lung and heart were removed, further fixed in 4% PFA for ≥2 h, and processed for paraffin embedding.

Paraffin embedded lungs were cut into 4 μm sections for immunohistochemistry (IHC). Expression of the contractile marker α-smooth muscle actin (α-SMA) or ET_A_ (endothelin receptor) were detected after antibody incubations by biotinylation with peroxidase. IHC sections were scanned on an Aperio Scanscope (ePathology Solutions) and analyzed by Aperio positive pixel count algorithm as intensity of strong positive staining divided by area (μm^2^) (25).

4% PFA-fixed hearts were cut in the mid-coronal plane before paraffin embedding. Heart paraffin blocks were then sectioned at 4μm thickness. Sirius Red staining for tissue collagen was performed as previously described (40). Briefly sections were incubated in 0.1% Sirius Red in saturated picric acid (Sigma-Aldrich) for 15 min, dehydrated and mounted in Depex and subjected to systematic digital analysis of entire sections using ImageJ software. Cardiac interstitial collagen content was quantified as a percentage of total myocardial area, excluding blood vessels. Quantitation was performed by an investigator blinded as to the identity of the samples using particle counting in ImageJ.

### Murine Protein Analysis

At day 5, lungs were harvested, washed in ice-cold PBS, snap frozen in liquid nitrogen and stored at −80°C. For analysis, the lungs were homogenized in lysis buffer (41) using an Ultra Turrax homogenizer. The homogenate was centrifuged for 10 min at 14,000 × g and the supernatants were assayed for protein. ELISAs for VEGF-A and endothelin-1 (R&D Systems) were performed according to the manufacturer's instructions. All results were normalized to total protein concentration by using the Pierce BCA Protein Assay (Thermo Fisher Scientific).

### Quantitative RT-PCR

At day 28, hearts were removed and washed in PBS and snap frozen in liquid nitrogen and stored at −80°C. Total RNA was isolated from hearts using TriReagent (Sigma-Aldrich Co. MO) and Ambion DNA-free DNA treatment used to remove contaminating DNA following the manufacturer's instructions. RNA was isolated using the RNA Mini Kit (Bioline), quantified with a NanoDrop (ND-100) spectrophotometer (Thermo Fisher Scientific), and assessed to have a 260:280 ratio of ~2.0. First strand cDNA synthesis was performed using the Life Technologies SuperScriptIII First-Strand Synthesis Kit (Invitrogen, MA). Heart RNA was analyzed by BioMark HD digital PCR (Fluidigm) using TaqMan primer probes listed in Supplementary Table 1. Gene expression values were normalized to the most stably expressed housekeeping gene, actin beta (*Actb*), across our samples. Relative expression was quantified using the ΔΔC_T_ method (42).

### *In vivo* Cine-Angiography and Surgical Preparation

At day 60 of age, mice were imaged via synchrotron radiation. For surgical preparation general anesthesia was induced with pentobarbital (1:10 diluted solution, 60 mg/kg). Subsequently, mice were intubated for artificial ventilation (6 μl/g tidal volume and ~170–190 breaths/min; AccuVent200 Small Animal Ventilator, Notting Hill Devices, Melbourne, Australia) and the right jugular vein was cannulated with 24-gauge Angiocath (Becton Dickinson, NJ, USA) following modification as previously described by Sonobe et al. (43), so that the tip of the catheter was placed in the right atrium or right ventricle. The right carotid artery was cannulated with a polyurethane catheter (Instech Solomon FunnelCath™ PUFC-C30-10) for arterial blood pressure and heart rate monitoring throughout experiments. Body temperature was maintained at 38°C with a thermostatically controlled heating pad. Anesthesia was maintained via additional intraperitoneal boluses of pentobarbital (20 mg/kg/h). Blood pressure was recorded via a disposable pressure transducer (MLR0699, AD Instruments, NSW, Australia) from the carotid arterial line. The signal was digitized at 1,000 Hz and recorded with CHART software (version 6.0, AD Instruments, NSW, Australia) to obtain mean arterial pressure (MAP) and heart rate (HR). The mouse was taped securely on a thin acrylic board in a supine position during surgery. Following surgical preparation, the board was then set in a vertical position in front of the Ruby X-ray detector.

X-rays at 34 keV (energy bandwidth 25–120 eV) and a flux of 6 × 10^11^ photons/mm^2^/s passed through the mouse chest and were recorded on X-ray detector (Imaging and Medical Beamline, Australian Synchrotron, Melbourne, Victoria, Australia) with a resolution of 16-bit at 30 ms intervals. High-resolution images were stored in a digital frame memory system with 1,024 × 1,024 pixel format with a 9.9 μm pixel size. Between recordings the X-ray beam was blocked with filters. The pulmonary angiograms were acquired with the long axis of the lung aligned vertically within the X-ray beam with an imaging field of ~10 × 10 mm. After verification of the placement, contrast agent was remotely injected through the jugular vein catheter as a bolus (90–120 μl over 2 s; Iomeron 350; Bracco-Eisai) using a syringe pump (PHD-2000, Harvard Apparatus, Holliston, MA, USA). Image acquisition was initiated right before iodine contrast injection and ~100 frames were recorded for each scan whilst ventilation was briefly interrupted at end inspiration to eliminate movement artifacts. After completion of the imaging protocol, animals were terminally anesthetized (pentobarbital, 100 mg/kg i.v).

Image analysis was performed with the use of ImageJ software (44). To eliminate background structure and enhance vessel visibility the frame obtained just before iodine injection was subtracted from frames acquired after injection. Subsequently angiograms were median filtered (2-pixel radius) for clarity. Each vessel was manually marked with a color-coded dot (green-1st generation, yellow-2nd, and orange-3rd) and all dots were counted automatically by ImageJ.

### Statistical Analysis

Data sets (raw data) were first tested for normality and equal variance (*P*-value to reject = 0.05), then analyzed by GraphPad Prism 7 (GraphPad Software) with one-way analysis of variance (ANOVA) and Tukey's multiple comparisons test. *In vivo* synchrotron angiography and TPV/RVET were analyzed by Student's *t*-test. Expression of α-SMA and ET_A_ were measured as strongly positive pixel count/area. For concentration-response curves, contraction was calculated as % reduction in initial artery area, and pEC_50_ and maxima obtained from non-linear regression curve fit using Prism. For all measures, data was expressed as mean ± standard error of the mean (SEM) and considered significantly different when *P* < 0.05.

## Results

### IL-1Ra Restores the Pulmonary Vascular Structure Impaired by Antenatal LPS and 65% O_2_

**Figure 1:**
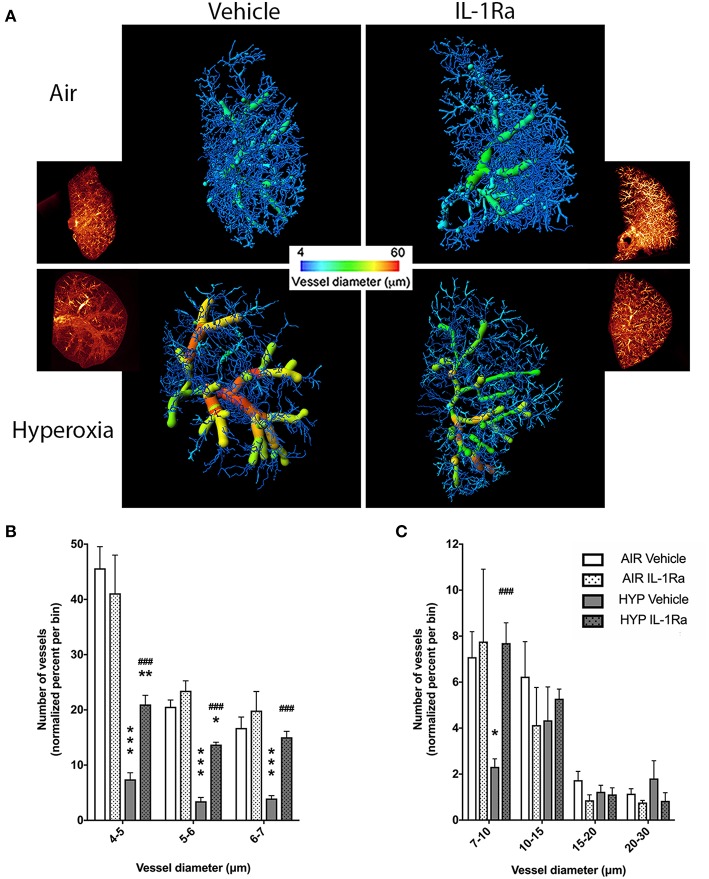
Pulmonary vascular injury caused by perinatal inflammation and postnatal hyperoxia is rescued by IL-1Ra. Pregnant C57BL6/J dams were injected i.p. with LPS at day 14 of gestation. Within 24 h after birth, pups were randomized to either 65% O_2_ (hyperoxia) or 21% O_2_ (room air). Pups also received daily s.c. injections of IL-1Ra or vehicle. At day 28, lungs were fixed and stained, and micro-CT imaging was performed. **(A)** One representative image of each group is shown; inset shows reconstructed lung volumes and larger image shows the filament filling color-coded for diameter size. Quantification of the number of vessels in the lung grouped by vessel diameter: **(B)** small vessels, 4–7 μm and **(C)** medium vessels, 7–30 μm. Vessel number was normalized to percent of total vessels per bin. Data are shown as mean ± SEM. *n* = 3–10 per group. *P < 0.05, **P < 0.01, and ***P < 0.001 for air vehicle vs. hyperoxia vehicle; ^*###*^*P* < 0.001 for hyperoxia vehicle vs. hyperoxia IL-1Ra.

Our clinically relevant double-hit BPD/BPD-PH model combines perinatal inflammation and 28 days of postnatal hyperoxia which leads to a severe BPD-like lung disease with a pronounced change in lung morphology as seen in BPD infants. Having shown in our model that IL-1Ra prevents the loss of alveoli that is characteristic of BPD (19, 26), the aim of the current study was to determine if blocking inflammation with IL-1Ra for 28 days ameliorates rarefaction of the pulmonary vascular bed in mice with BPD and thereby also reduces pulmonary hypertension. By using high resolution *ex vivo* X-ray micro CT scanning at the Australian Synchrotron, we quantified the number of pulmonary vessels in the right superior pulmonary lobe of day 28 mice (Figure 1A). To illustrate the distribution of blood vessel numbers by diameter as well as the relative abundance of vessels, the vessel numbers were converted into percentages binned by diameter, then normalized to the air vehicle group (Figures 1B,C). Our results revealed a marked reduction in the number of microvessels in the lung from pups reared in hyperoxia compared to pups housed in room air for 28 days (e.g., −84% for capillaries sized 4–5 μm in diameter, −83% for 5–6 μm, −76% for 6–7 μm; Figure 1B). Treatment with IL-1Ra conferred significant protection from BPD-PH-associated loss in the small and medium vessels (Figures 1A–C). In summary, our findings suggest that if commenced early in life, daily treatment with IL-1Ra protects the pulmonary vascular bed from the injury induced by perinatal LPS and postnatal hyperoxia.

### IL-1Ra Improves Pulmonary Vascular Resistance: Physiological Function and Molecular Aspects

**Figure 2:**
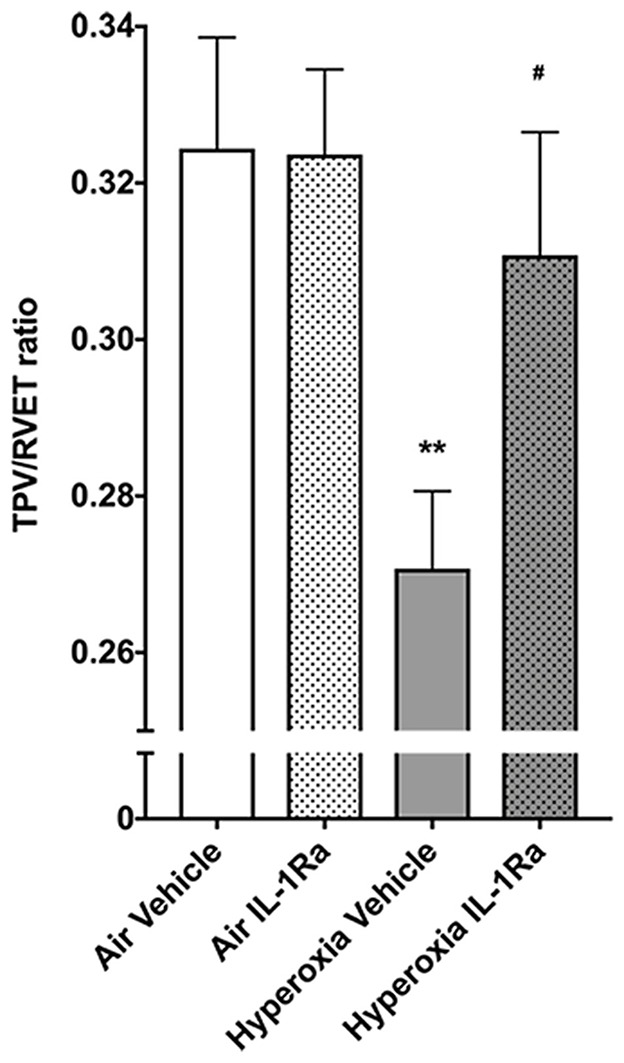
Mice treated with IL-1Ra are protected from the BPD-PH-associated increase in pulmonary vascular resistance. Echocardiography was performed on each of the experimental groups on day 28 of life in the same animals shown in Figure 1. TPV/RVET ratio, an index of RV function, was measured; *n* = 12–20 per group. Data are shown as mean ± SEM; **P < 0.01 for air vehicle vs. hyperoxia vehicle; ^#^*P* < 0.05 for hyperoxia vehicle vs. hyperoxia IL-1Ra.

The changes we observed in the number of vessels in the pulmonary vascular bed at 28 days of life in BPD mice begged the question whether this tissue injury had functional consequences. Accordingly, we subjected the same mice that subsequently underwent synchrotron analysis to non-invasive echocardiography, the diagnostic tool of choice in preterm infants for diagnosing pulmonary hypertension (45). Specifically, we assessed pulmonary vascular resistance in 28 days-old mice using the surrogate parameter time-to-peak-velocity/right ventricular ejection time (TPV/RVET) ratio, where the TPV/RVET ratio is inversely related to pulmonary vascular resistance. We found a significant reduction in TPV/RVET from 0.32 in pups housed in room air to 0.27 in the hyperoxia control group (Figure 2). The reduction in TPV/RVET caused by hyperoxia was completely prevented in hyperoxic pups receiving daily treatment with IL-1Ra, which had a TPV/RVET ratio of 0.31, similar to the room air control pups (Figure 2).

**Figure 3:**
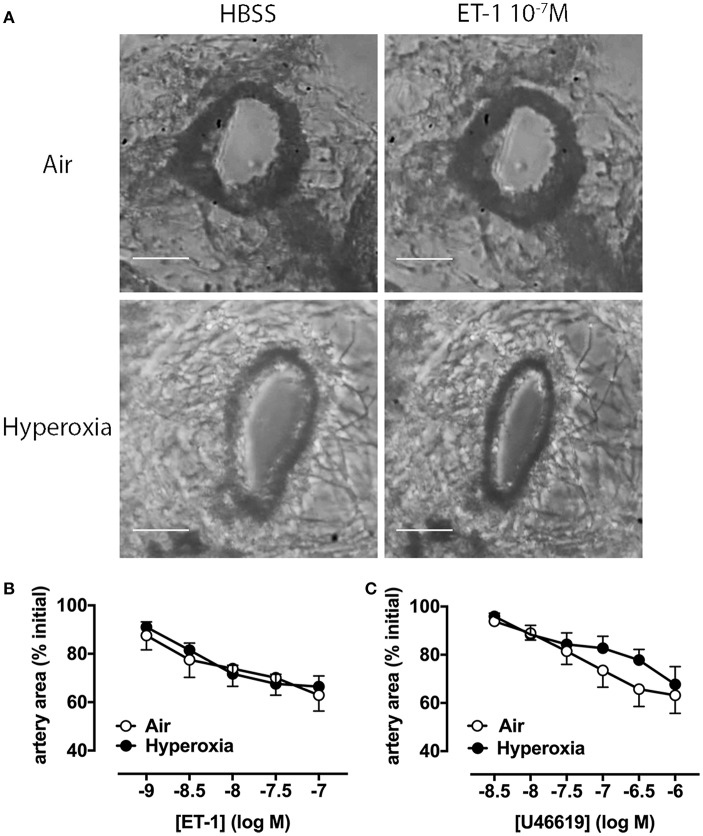
Pulmonary artery contraction in response to ET-1 and U46619 is not altered by hyperoxia in PCLS. Pulmonary artery reactivity was assessed in PCLS prepared from 28 days-old mice after exposure to antenatal LPS and postnatal hyperoxia. **(A)** One representative image per group is depicted, showing an artery during perfusion with HBSS (i.e., vehicle) or ET-1. Concentration-response curves for ET-1 **(B)** and U46619 **(C)** are expressed as percent initial artery area; scale bars are 100 μm. Data are shown as mean ± SEM, *n* = 7–9 per group.

We then assessed whether additional mechanisms beyond vascular rarefaction could be contributing to this hyperoxia-induced increase in pulmonary vascular resistance we observed *in vivo*. To determine if pulmonary artery reactivity to vasoconstrictors was increased by hyperoxia, we initially performed *ex vivo* experiments using precision-cut lung slices (PCLS) from either 28 days-old room air or hyperoxia-housed pups, without IL-1Ra treatment. We visualized intrapulmonary artery responses to endothelin-1 (ET-1), a key contractile mediator often elevated in pulmonary arterial hypertension, and to the thromboxane mimetic U46619 (Figure 3).

Representative images show that a maximally effective concentration of ET-1 (10^−7^ M) reduces luminal area by ~40% in arteries from both air and hyperoxia groups (Figure 3A). Averaged data show reductions in artery area during perfusion with increasing ET-1 or U46619 concentrations at 10 min intervals (Figures 3B,C). A representative video of PCLS prepared from a hyperoxic mouse, showing intrapulmonary artery constriction to ET-1 (1–100 nM), with increasing concentrations added at 10 min intervals, where 1 s corresponds to 2 min in real time has been provided in the supplementary material (Supplementary Video 1; the airway is shown on the left, the artery on the right). In PCLS from air control mice, ET-1 was ~10-fold more potent than U46619, but both agonists caused a similar maximum % reduction in artery luminal area (ET-1 43 ± 6%, *n* = 8; U46619 38 ± 8%, *n* = 8). Interestingly, neither the potency of either agonist, nor its maximum response, was significantly altered by hyperoxia (Figures 3B,C), suggesting that hyperoxia does not alter vascular reactivity.

**Figure 4:**
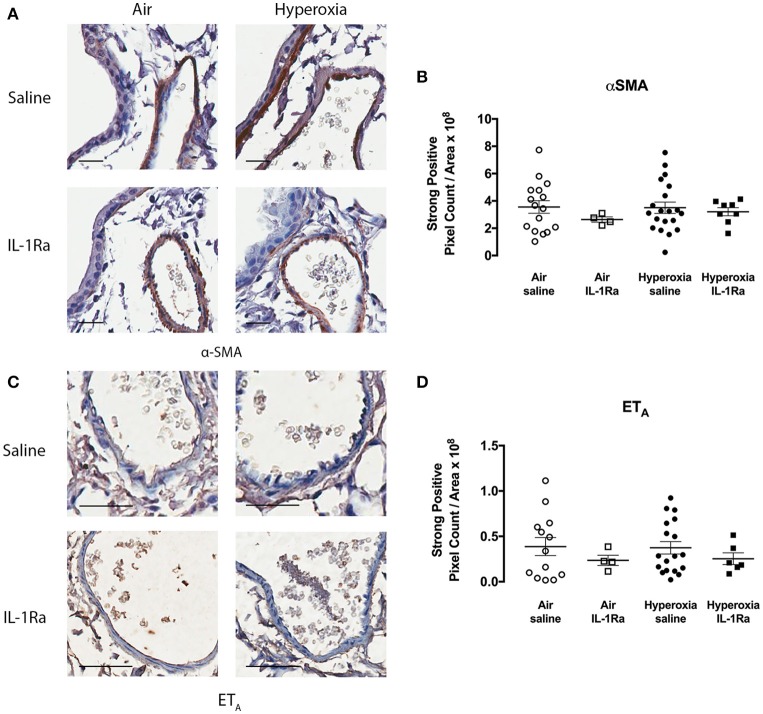
α-SMA and ET_A_ abundance in pulmonary arteries is not altered following hyperoxia. Immunohistochemistry for α-SMA and ET_A_ was performed in lung sections from the air vehicle and hyperoxia vehicle groups. Each representative image for α-SMA **(A)** shows an airway on the left and an artery on the right. Scale bars = 100 μm. Representative images for ET_A_ **(C)** show an artery only. Scale bar = 50 μm. Staining was analyzed using the Aperio positive pixel count algorithm and expressed as intensity of strongly positive staining divided by area (μm^2^) for α-SMA **(B)** and ET_A_ **(D)**. Data are shown as mean ± SEM, *n* = 13–20 mice per group.

In addition, we assessed whether hyperoxia altered the abundance of vascular smooth muscle (determined by measuring α-SMA), since an increase in smooth muscle has the potential to augment vasoconstriction and thus pulmonary artery resistance *in vivo*. Given that we observed increases in α-SMA staining in the adjacent airways of pups exposed to hyperoxia (25), we were somewhat surprised to find no difference in α-SMA staining by immunohistochemistry in the pulmonary arteries between air and hyperoxia groups (Figures 4A,B). IL-1Ra treatment also did not alter α-SMA abundance in the pulmonary arteries in both air and hyperoxia, when compared to air vehicle mice. We also investigated the receptor ET_A_, which mediates contraction in response to ET-1. No alterations in ET_A_ abundance on vascular smooth muscle could be observed in the hyperoxia vehicle, and IL-1Ra-treated air and hyperoxia groups, when compared to air vehicle (Figures 4C,D).

Perinatal inflammation and postnatal exposure to 65% O_2_ resulted in a significant increase in pulmonary vascular resistance *in vivo*, but we found neither an increase in vascular smooth muscle abundance, nor a change in the constrictive response of pulmonary arteries *ex vivo*, nor altered abundance of ET_A_ in the pulmonary arteries. This being the case, the effects of IL-1Ra treatment on these parameters were not explored further. Instead, we focused on the earlier timepoint of day 5, at which molecular changes occur that affect the development of the pulmonary vascular bed (11).

**Figure 5:**
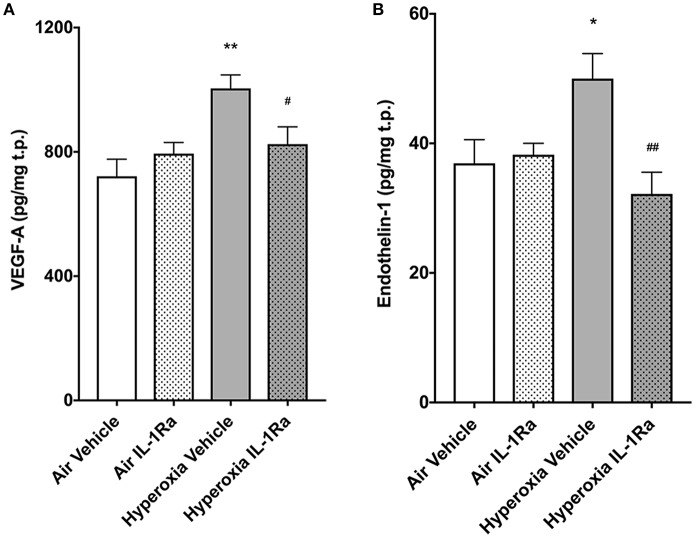
IL-1Ra prevents increases in VEGF and ET-1 on day 5 of BPD-PH. The abundance of VEGF-A **(A)** and ET-1 **(B)** was determined by ELISA in d5 lungs exposed to prenatal LPS and postnatal hyperoxia or room air. Data shown as means normalized to total protein (t.p.) ± SEM. *n* = 9–13 per group; *P < 0.05 and **P < 0.005 for room air vehicle vs. hyperoxia vehicle; ^#^*P* < 0.05 and ^*##*^*P* < 0.005 for hyperoxia vehicle vs. hyperoxia IL-1Ra.

Murine lungs on experimental day 5 were assessed for protein abundance of vascular endothelial growth factor (VEGF) and ET-1, mediators known to affect pulmonary angiogenesis. We found that exposure to 65% O_2_ moderately, but significantly, increased VEGF-A (1.4-fold, Figure 5A), the member of the VEGF family with the greatest impact on physiological and pathophysiological functions in the lung (21). Moreover, ET-1 increased 1.4-fold (Figure 5B) in hyperoxia vehicle pups compared to room air vehicle pups. Blockade of inflammation by daily injections of IL-1Ra over 5 days prevented the increase in VEGF-A and ET-1 (Figures 5A,B).

In summary, daily treatment of BPD mice with IL-Ra improves vascular resistance at day 28 of life, exerts a beneficial effect on early life vascular markers and restores subsequent vascular development.

### IL-1Ra Protects From Cardiac Inflammation

**Figure 6:**
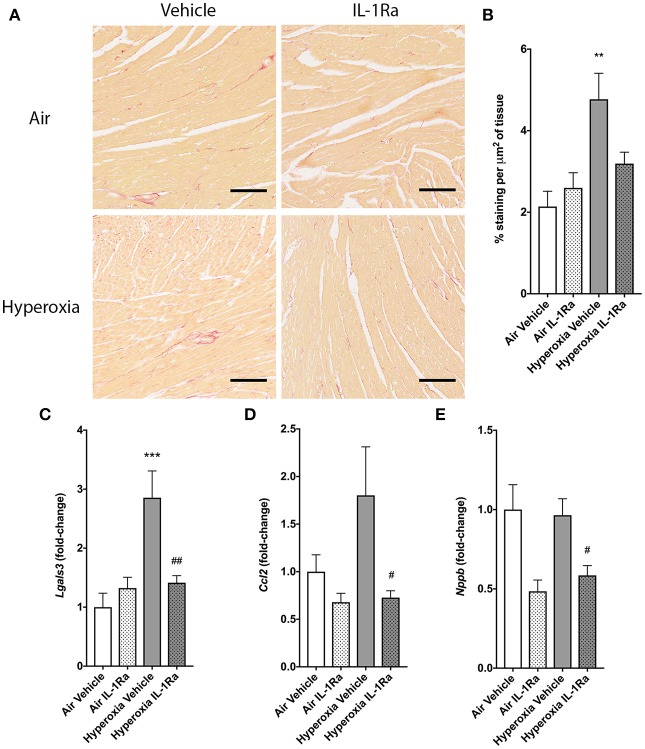
Effects of IL-1Ra on the heart in murine BPD-PH. At day 28 of the BPD-PH model, hearts were stained with Sirius Red and whole heart slices were analyzed. **(A)** One representative image per group is depicted. Scale bars, 100 μm; *n* = 7–10 per group. **(B)** Quantification of Sirius Red staining in *n* = 7–10 pups per group. Data shown as mean ± SEM. **(C–E)** Real-time PCR was performed on whole heart homogenates for **(C)** *Lgals3*, **(D)** *Ccl2* and **(E)** *Nppb*. Results were normalized to *Actb* and depicted as fold-change relative to the lowest expressed gene ± SEM. **P < 0.01 and ***P < 0.001 for air vehicle vs. hyperoxia vehicle; ^#^*P* < 0.05 and ^*##*^*P* < 0.005 for hyperoxia vehicle vs. hyperoxia IL-1Ra.

To evaluate the impact of perinatal LPS and hyperoxia on myocardial remodeling and function, we next assessed left ventricular (LV) function by echocardiography and a set of established markers for cardiac inflammation and fibrosis at 28 days. The left ventricle diameter at the end of diastole (LVIDd) and systole (LVIDs) showed no change in the presence of hyperoxia or IL-1Ra (Supplementary Figures 1A,B). Fractional shortening was calculated as a measure of LV function and similarly did not show regulation by either hyperoxia or IL-1Ra (Supplementary Figure 1C). Given that inflammation is associated with structural remodeling in many tissues, including the heart, we also investigated cardiac fibrosis, as measured by Sirius Red-stained collagen in whole hearts (46, 47). Fibrosis was significantly, 2.2-fold increased in mice exposed to hyperoxia in comparison with their counterparts reared in room air (Figures 6A,B). Daily treatment with IL-1Ra reduced collagen abundance in the hyperoxia group, although this difference failed to reach significance (*P* = 0.054). In addition, we assessed changes in the expression of markers of cardiac inflammation and fibrotic remodeling by RT-PCR. *Lgals3*, which encodes the protein Mac-2/galectin-3, is predominantly expressed by activated macrophages and known to regulate inflammatory and fibrotic responses in the heart (48), was markedly increased (2.9-fold) by exposure to hyperoxia when compared to air vehicle. This increase was reduced by 50% in daily IL-1Ra-treated hyperoxic mice (Figure 6C).

Consistent with the data for *Lgals3* expression, the macrophage recruitment chemokine (C-C motif) ligand 2 (CCL2, also called MCP-1) was also, albeit non-significantly, increased 1.8-fold by hyperoxia, and *Ccl2* expression was significantly lower (-59%) in hearts continuously exposed to hyperoxia but treated with IL-1Ra (Figure 6D). B-type natriuretic peptide (BNP; gene name *Nppb*) is released by cardiomyocytes in response to stretch caused by increased ventricular blood volume. Although hyperoxia did not increase *Nppb* expression compared to hearts from air-breathing controls, treatment with IL-1Ra reduced *Nppb* expression in both air and hyperoxia (39% decrease compared to hyperoxia vehicle, Figure 6E).

Taken together, these data suggest that prenatal inflammation accompanied by postnatal hyperoxia do not modify LV function at day 28 but have a detrimental effect on the myocardium.

### Early Life IL-1Ra Treatment Confers Long-Term Benefits on the Pulmonary Vasculature

**Figure 7:**
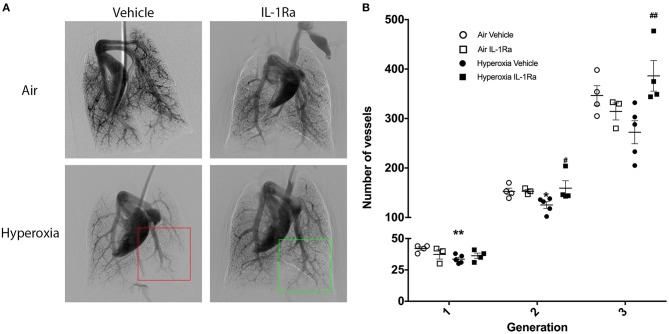
Sustained treatment effects of IL-1Ra on day 60 of life. The pulmonary vasculature of 60 days-old BPD-PH mice was visualized by cine-angiography. **(A)** One representative synchrotron radiation angiogram of the pulmonary vasculature per group is depicted. The red squares highlight poor regional perfusion of distal blood vessels in hyperoxia vehicle mice and the green square shows the amelioration of vascular development afforded by IL-1Ra. **(B)** Quantification of absolute blood vessel number; *n* = 3–5 mice per group. Data are mean ± SEM. *P < 0.05 and **P < 0.005 for air vehicle vs. hyperoxia vehicle; ^#^*P* < 0.05 and ^*##*^*P* < 0.005 for hyperoxia vehicle vs. hyperoxia IL-1Ra.

Next, we investigated whether the beneficial effects of IL-1Ra on the pulmonary vascular bed lasted beyond day 28. A 60 day experimental endpoint was selected, representing early adulthood, i.e., ~20 years of age, in the human (49). We ceased IL-1Ra treatment on day 28, housed pups of the four experimental groups in normal husbandry conditions until day 60, then performed *in vivo* cine-angiography at day 60. We observed that the vascular development was substantially compromised in hyperoxia vehicle adult mice when compared to air vehicle mice (Figure 7A). A video of the live *in vivo* cine-angiography for a representative hyperoxia vehicle mouse at day 60 is provided as supplementary material (Supplementary Video 2; the video first shows the cine-angiography in real time, then replayed at one third of the speed). Early life treatment with IL-1Ra ameliorated this vascular growth arrest: the green square in Figure 7A highlights a well-developed distal vascular region of a 60 days-old mouse treated daily with IL-1Ra for 28 days, which compares favorably to the red square, indicating a region with poor perfusion of distal blood vessels in a representative hyperoxia vehicle pup. Quantification of the overall visible vessel number (Supplementary Figure 2) revealed that in each branching generation vessel numbers were markedly reduced in the hyperoxia vehicle group (−18% in generation 2 to −21% in generation 3, Figure 7B). IL-1Ra rescued the number of pulmonary blood vessels when compared to vehicle-treated hyperoxia pups (Figure 7B) in generations 2 and 3 (30 and 48%).

Thus, the pulmonary vascular injury induced by perinatal inflammation and 28 days of hyperoxia in our murine BPD model persists into early adulthood. Importantly, early life treatment with IL-1Ra confers lasting protection after treatment has ceased.

## Discussion

Previous work by us (19, 26) and others (50), showed the protective properties of IL-1Ra on alveologenesis in a murine model of BPD. In addition, we also observed that overall number of CD45^+^ immune cells was up to three times lower in the hyperoxia group when compared to air controls and there was increased activation of macrophages and DCs (CD11b^+^ and GR1^+^ cells); IL-1Ra treatment partially restored these values back to normal. Here, we set out to investigate the effects of perinatal LPS and postnatal hyperoxia on the pulmonary vasculature and the heart of the neonatal mouse, as well as potential beneficial actions of IL-1Ra in preventing the development of BPD-PH (51). The major findings in our preclinical model of BPD-PH are that prophylaxis with IL-1Ra prevents the increase in pulmonary vascular resistance *in vivo* and limits the loss of pulmonary vascular density at both day 28 and 60 of life. Additionally, we revealed that IL-1Ra reduces VEGF and ET-1 at day 5 in the lung. Moreover, we demonstrated that treatment with IL-1Ra reduces the fibrotic effect observed in the heart in BPD-PH.

To the best of our knowledge, our study is the first to demonstrate a beneficial effect of IL-1Ra on vascular resistance *in vivo* in the setting of experimental BPD-PH, as exemplified by echocardiography improving the TPV/RVET ratio. In contrast to the marked changes in RV function that reflect pulmonary vascular dysfunction, LV function (fractional shortening) was not changed at 28 days, highlighting the selective impact of BPD on the RV at this stage of the disease. These data add substantially to the evidence implicating IL-1 in the pathogenesis of PH in animals and humans (52–56). Moreover, studies investigating IL-1Ra as a treatment option for PH are sparse. In an adult rat inflammatory PH model, daily IL-1Ra treatment brings about a significant reduction of PH (57), and neonatal piglets have been shown to be protected from neonatal PH when treated with IL-1Ra (58). A recent single-arm, open label study has reported IL-1Ra successfully treats human adult PH (59).

Exposure to high oxygen concentrations and to mechanical ventilation leads to an early rise in VEGF, followed by a decline in both animals and humans developing BPD (60–64). Prenatal chronic conditional overexpression of VEGF-A in the murine lung increases early life mortality, causes alveolar remodeling, and increases inflammation (65). Based on these studies, we focused our investigation on VEGF-A and found it to be increased on day 5 in murine BPD lungs when compared to air lungs. In IL-1Ra-treated hyperoxic pups, VEGF-A abundance was similar to that in control air pups. Hence, elevated VEGF-A may contribute to the disruption of early life pulmonary vascular development, and IL-1Ra reverses this effect. Our findings of early elevated VEGF moreover resemble neonatal hyperoxia studies in rodents as well as data in human infants, showing that regulation of VEGF isoforms and its receptors in hyperoxia is dependent on timing and oxygen concentrations (60–63).

It is well-known that pro-inflammatory cytokines including IL-1β increase VEGF abundance in a variety of cell types, including lung cells (66–69). Furthermore, several lines of evidence implicate IL-1 in BPD. A study in 1,062 preterm infants (70), as well as smaller studies (71–73), report an association between BPD or death and increased abundance of IL-1β. Additionally, IL-1Ra modulates endothelial cell (EC) proliferation (74) and thereby might contribute to EC protection, re-growth and vessel healing after vascular injury. In more general terms, increased abundance of IL-1 in early lung development induces pulmonary VEGF production and secretion, thereby promoting dysangiogenesis. Our findings suggest that vascular remodeling can be prevented by immediate IL-1Ra treatment after birth, just as we reported previously for alveolar changes in BPD (26).

ET-1 plays an important role in maintaining normal pulmonary circulation in early life and is known to be elevated by inflammation and hyperoxia in lung and plasma (75, 76) in rodent models of disease. ET-1 also increases microvascular permeability *ex vivo* in rat lungs (77). Abnormally elevated ET-1 has been studied in plasma and tracheobronchial aspirates of infants suffering from BPD-PH for more than two decades (20, 78, 79). These findings are consistent with our data showing increased ET-1 protein in day 5 lungs exposed to antenatal inflammation and postnatal hyperoxia. Here, we show that IL-1Ra treatment restored ET-1 in lung lysates back to air control levels. Since ET-1 is known to be increased directly and indirectly via IL-1 (80), we infer that one of the mechanisms of IL-1Ra function is to block the induction of ET-1 by IL-1. Considering the data available, we conclude that IL-1Ra protects newborn mice from the dysangiogenesis promoted by ET-1 and VEGF-A immediately after birth.

We assessed the involvement of increased vascular reactivity in BPD-PH by measuring the reactivity of intrapulmonary arteries *ex vivo* using PCLS from pups on day 28 of life. We have previously used this approach to establish that airway contraction in response to methacholine *ex vivo* is increased in PCLS from the same model (25). However, although in the current study vascular density and pulmonary vascular resistance were altered by hyperoxia *in vivo*, there was no difference in *ex vivo* vasoconstriction to ET-1 or U46619. Consistent with these findings, the abundance of ET_A_ receptors that mediate the effects of ET-1 on vascular tone was not altered, and α-SMA, a marker of remodeling of the pulmonary arteries associated with increased constriction, was not elevated. Other studies have shown that isolated pulmonary arteries from BPD rats exhibit increased vasoreactivity to U46619 (81) and in a 14 day BPD rat model increased vasoconstriction was associated with PH (82). It is possible that the absence of an increase in arterial α-SMA in hyperoxic pulmonary arteries in our mouse model could explain these discrepancies. However, increased α-SMA associated with vascular smooth muscle thickening in pulmonary arteries of hyperoxic mice was also reported in a study which had applied 85% O_2_ (83). We have shown that 85% O_2_ represents a more severe model of BPD (19) when compared to the 65% O_2_ we applied here; thus, a lower O_2_ level could account for the absence of *ex vivo* vascular reactivity and markers of vascular remodeling in our study.

The molecular mechanisms associated with right ventricular dysfunction and failure in BPD-PH are poorly understood, and therapeutic options are limited. Hence, we also investigated effects of our BPD model on tissue remodeling processes in the heart. Despite no change in LV function or dimensions being detected, our data show that exposure to LPS and hyperoxia induces tissue fibrosis and that IL-1Ra reversed these changes, indicating an important contribution by IL-1 to the fibrotic process (84). *Lgals3* and *Ccl2* have both been implicated in a variety of processes associated with heart failure, including myofibroblast proliferation, fibrogenesis, tissue repair, inflammation, and ventricular remodeling (85, 86), and both were therefore investigated in our model. Our mechanistic studies indicate that *Lgals3* and Ccl2 were both induced by hyperoxia and significantly reduced by daily treatment with IL-1Ra, pointing to an IL-1-dependent mechanism. These data support the concept that neonatal hearts share some common inflammatory pathways with adult hearts to promote cardiac tissue remodeling (87).

Our data showed that cardiac *Nppb* expression was not increased by hyperoxia, but IL-1Ra was able to reduce mRNA expression in both air and hyperoxia. Given that there were no changes in cardiac dimensions in any group, as we measured by echocardiography, our data suggest that IL-1Ra treatment might only reverse LPS-induced expression of *Nppb* in the 28 day heart as all groups were exposed to antenatal inflammation at E14. It is important to note that whole hearts were used for analysis and therefore potential differences between the left and right ventricle could not be detected. However, inflammatory mediators can induce BNP mRNA expression (*Nppb*). Binding of LPS to its receptor and stimulation of downstream pathways, including p38MAPK activation and induction of RAC1 and GATA elements, results in transactivation of the BNP promoter (88). Other studies report that TNF and IL-1β can selectively stimulate BNP at the transcriptional and translational levels in cardiomyocytes (89). Therefore, we can conclude that IL-1Ra treatment improves antenatal LPS induced long term *Nppb* expression and potential hemodynamic changes in the 28 day old heart.

In summary, previous studies and our own results point to the conclusion that decreases in vascular density and therefore an increase in vascular resistance, rather than changes in vascular reactivity, could be the cause of BPD-PH. This finding could explain why treatment of BPD-PH with vasodilators has shown limited efficacy in preventing BPD and/or BPD-PH in human infants (90), and therapeutic efforts to restore the vascular density of the lung should also be considered. IL-1Ra exerts beneficial effects by blocking IL-1 and indirectly by inhibiting VEGF and ET-1, thereby improving pulmonary vascular density and pulmonary vascular resistance. Although further research is needed to prove these hypotheses, we suggest that IL-1Ra could represent a supportive therapeutic to restore pulmonary homeostasis in neonatal cardiopulmonary disease.

## Data Availability

All datasets generated for this study are included in the manuscript and/or the Supplementary Files.

## Ethics Statement

The animal studies, imaging experiments, and protocols were carried out in accordance with the recommendations and approvals of the Animal Review Board MMCA of Monash University and the Australian Synchrotron animal ethics committee.

## Author Contributions

All authors were involved in drafting the article or revising it critically for important intellectual content, and all authors approved the final version to be published. CB and CN-P had full access to all of the data in the study and take responsibility for the integrity of the data and the accuracy of the data analysis. JP, MN, and CN-P study conception and design. CB, MKo, EL, KE, AS, IR, DS, HT, MKr, SC, AM, JP, and CN-P acquisition of data. CB, MKo, EL, KE, AM, PB, MY, JB, JP, MN, and CN-P analysis and interpretation of data. MC, JB, JP, and CN-P supervision. JB, JP, MN, and CN-P funding acquisition.

### Conflict of Interest Statement

The authors declare that the research was conducted in the absence of any commercial or financial relationships that could be construed as a potential conflict of interest.

## Supplementary Material

Supplementary Video 1 Video of PCLS prepared from a hyperoxic mouse, showing intrapulmonary artery constriction to ET-1 (1–100 nM), with increasing concentrations added at 10 min intervals. The airway is shown on the left and the artery is shown on the right. One second corresponds to 2 min in real time.

Supplementary Video 2 Video of the live *in vivo* cine-angiography for a representative hyperoxia vehicle mouse at day 60. Videos depict cine-angiography in real time and then replayed at one third of the speed.

## References

[1] Van Marter LJ. Epidemiology of bronchopulmonary dysplasia. Semin Fetal Neonatal Med. (2009) 14:358–66. 10.1016/j.siny.2009.08.007 19783238

[2] Stoll BJ Hansen NI Bell EF Shankaran S Laptook AR Walsh MC. Neonatal outcomes of extremely preterm infants from the NICHD Neonatal Research Network. Pediatrics. (2010) 126:443–56. 10.1542/peds.2009-2959 20732945 PMC2982806

[3] Fanaroff AA Stoll BJ Wright LL Carlo WA Ehrenkranz RA Stark AR. Trends in neonatal morbidity and mortality for very low birthweight infants. Am J Obstet Gynecol. (2007) 196:147 e141–8. 10.1016/j.ajog.2006.09.014 17306659

[4] Schmidt B Roberts RS Davis PG Doyle LW Asztalos EV Opie G. Prediction of late death or disability at age 5 years using a count of 3 neonatal morbidities in very low birth weight infants. J Pediatr. (2015) 167:982–6 e982. 10.1016/j.jpeds.2015.07.067 26318030

[5] Speer CP Pulmonary inflammation and bronchopulmonary dysplasia. J Perinatol. (2006) 26 (Suppl. 1):S57–62; discussion S63–54. 10.1038/sj.jp.7211476 16625227

[6] Halvorsen T Skadberg BT Eide GE Roksund OD Carlsen KH Bakke P. Pulmonary outcome in adolescents of extreme preterm birth: a regional cohort study. Acta Paediatr. (2004) 93:1294–300. 10.1111/j.1651-2227.2004.tb02926.x 15499947

[7] Aukland SM Rosendahl K Owens CM Fosse KR Eide GE Halvorsen T. Neonatal bronchopulmonary dysplasia predicts abnormal pulmonary HRCT scans in long-term survivors of extreme preterm birth. Thorax. (2009) 64:405–10. 10.1136/thx.2008.103739 19158126

[8] Bhat R Salas AA Foster C Carlo WA Ambalavanan N. Prospective analysis of pulmonary hypertension in extremely low birth weight infants. Pediatrics. (2012) 129:e682–9. 10.1542/peds.2011-1827 22311993 PMC3289526

[9] Khemani E Mcelhinney DB Rhein L Andrade O Lacro RV Thomas KC. Pulmonary artery hypertension in formerly premature infants with bronchopulmonary dysplasia: clinical features and outcomes in the surfactant era. Pediatrics. (2007) 120:1260–9. 10.1542/peds.2007-0971 18055675

[10] Taglauer E Abman SH Keller RL. Recent advances in antenatal factors predisposing to bronchopulmonary dysplasia. Semin Perinatol. (2018) 42:413–24. 10.1053/j.semperi.2018.09.002 30389227 PMC6286866

[11] Alvira CM. Aberrant pulmonary vascular growth and remodeling in bronchopulmonary dysplasia. Front Med. (2016) 3:21. 10.3389/fmed.2016.00021 27243014 PMC4873491

[12] Stenmark KR Abman SH. Lung vascular development: implications for the pathogenesis of bronchopulmonary dysplasia. Ann Rev Physiol. (2005) 67:623–61. 10.1146/annurev.physiol.67.040403.102229 15709973

[13] Farquhar M Fitzgerald DA. Pulmonary hypertension in chronic neonatal lung disease. Paediatr Respir Rev. (2010) 11:149–53. 10.1016/j.prrv.2010.05.001 20692628

[14] Kim GB. Pulmonary hypertension in infants with bronchopulmonary dysplasia. Korean J Pediatr. (2010) 53:688–93. 10.3345/kjp.2010.53.6.688 21189939 PMC2994133

[15] Berkelhamer SK Mestan KK Steinhorn RH. Pulmonary hypertension in bronchopulmonary dysplasia. Semin Perinatol. (2013) 37:124–31. 10.1053/j.semperi.2013.01.009 23582967 PMC4464837

[16] Sehgal A Malikiwi A Paul E Tan K Menahem S. Systemic arterial stiffness in infants with bronchopulmonary dysplasia: potential cause of systemic hypertension. J Perinatol. (2016) 36:564–9. 10.1038/jp.2016.10 26914016

[17] Hassoun PM Mouthon L Barbera JA Eddahibi S Flores SC Grimminger F. Inflammation, growth factors, and pulmonary vascular remodeling. J Am College Cardiol. (2009) 54:S10–19. 10.1016/j.jacc.2009.04.006 19555853

[18] Price LC Wort SJ Perros F Dorfmuller P Huertas A Montani D. Inflammation in pulmonary arterial hypertension. Chest. (2012) 141:210–21. 10.1378/chest.11-0793 22215829

[19] Nold MF Mangan NE Rudloff I Cho SX Shariatian N Samarasinghe TD. Interleukin-1 receptor antagonist prevents murine bronchopulmonary dysplasia induced by perinatal inflammation and hyperoxia. Proc Natl Acad Sci USA. (2013) 110:14384–9. 10.1073/pnas.1306859110 23946428 PMC3761642

[20] Niu JO Munshi UK Siddiq MM Parton LA. Early increase in endothelin-1 in tracheal aspirates of preterm infants: correlation with bronchopulmonary dysplasia. J Pediatr. (1998) 132:965–70. 10.1016/S0022-3476(98)70392-0 9627587

[21] Voelkel NF Vandivier RW Tuder RM. Vascular endothelial growth factor in the lung. Am J Physiol Lung Cell Mol Physiol. (2006) 290:L209–21. 10.1152/ajplung.00185.2005 16403941

[22] Walsh MC Szefler S Davis J Allen M Van Marter L Abman S. Summary proceedings from the bronchopulmonary dysplasia group. Pediatrics. (2006) 117:S52–6. 10.1542/peds.2005-0620I 16777823

[23] Mcevoy CT Jain L Schmidt B Abman S Bancalari E Aschner JL. Bronchopulmonary dysplasia: NHLBI workshop on the primary prevention of chronic lung diseases. Ann Am Thorac Soc. (2014) 11 (Suppl. 3):S146–53. 10.1513/AnnalsATS.201312-424LD 24754823 PMC4112507

[24] Dinarello CA Simon A Van Der Meer JW. Treating inflammation by blocking interleukin-1 in a broad spectrum of diseases. Nat Rev Drug Discov. (2012) 11:633–52. 10.1038/nrd3800 22850787 PMC3644509

[25] Royce SG Nold MF Bui C Donovan C Lam M Lamanna E Airway remodeling and hyperreactivity in a model of bronchopulmonary dysplasia and their modulation by IL-1Ra. Am J Respir Cell Mol Biol. (2016) 55:858–68. 10.1165/rcmb.2016-0031OC 27482635

[26] Rudloff I Cho SX Bui CB Mclean C Veldman A Berger PJ. Refining anti-inflammatory therapy strategies for bronchopulmonary dysplasia. J Cell Mol Med. (2017) 21:1128–38. 10.1111/jcmm.13044 27957795 PMC5431131

[27] Ten Have-Opbroek AA Lung development in the mouse embryo. Exp Lung Res. (1991) 17:111–30. 10.3109/01902149109064406 2050021

[28] Backstrom E Hogmalm A Lappalainen U Bry K. Developmental stage is a major determinant of lung injury in a murine model of bronchopulmonary dysplasia. Pediatr Res. (2011) 69:312–8. 10.1203/PDR.0b013e31820bcb2a 21178818

[29] Fox J Barthold S Davisson M Newcomer C Quimby F Smith A The Mouse in Biomedical Research. Burlington, MA: Elsevier (2006).

[30] Gao XM Dart AM Dewar E Jennings G Du XJ. Serial echocardiographic assessment of left ventricular dimensions and function after myocardial infarction in mice. Cardiovasc Res. (2000) 45:330–8. 10.1016/S0008-6363(99)00274-6 10728353

[31] Metscher BD. MicroCT for comparative morphology: simple staining methods allow high-contrast 3D imaging of diverse non-mineralized animal tissues. BMC Physiol. (2009) 9:11. 10.1186/1472-6793-9-11 19545439 PMC2717911

[32] Stephenson RS Boyett MR Hart G Nikolaidou T Cai X Corno AF. Contrast enhanced micro-computed tomography resolves the 3-dimensional morphology of the cardiac conduction system in mammalian hearts. PLoS ONE. (2012) 7:e35299. 10.1371/annotation/1baecd19-92b6-4683-b7d7-39c13a3f2e15 22509404 PMC3324466

[33] Massive (2016). MASSIVE. DNA Integrated Communications. Available online at: https://www.massive.org.au/ (accessed July 5, 2017).

[34] Gureyev TE Nesterets Y Ternovski D Thompson D Wilkins SW Stevenson AW. (2011).Toolbox for advanced x-ray image processing, in Proceedings Volume 8141, Advances in Computational Methods for X-Ray Optics II; 81410B (San Diego, CA).

[35] Limaye A (2012). Drishti: a volume exploration and presentation tool, in Proceedings Volume 8506, Developments in X-Ray Tomography VIII; 85060X (San Diego, CA).

[36] Imagemagick Studio L (2017). ImageMagick. Available online at: https://imagemagick.org (accessed July 5, 2017).

[37] Maksimenko A (2017). ctas. Available online at: https://github.com/antonmx/ctas (accessed July 5, 2017).

[38] Schindelin J Arganda-Carreras I Frise E Kaynig V Longair M Pietzsch T. Fiji: an open-source platform for biological-image analysis. Nat Methods. (2012) 9:676–82. 10.1038/nmeth.2019 22743772 PMC3855844

[39] Bourke JE Bai Y Donovan C Esposito JG Tan X Sanderson MJ. Novel small airway bronchodilator responses to rosiglitazone in mouse lung slices. Am J Respir Cell Mol Biol. (2014) 50:748–56. 10.1165/rcmb.2013-0247OC 24188042 PMC4068922

[40] Rickard AJ Morgan J Tesch G Funder JW Fuller PJ Young MJ. Deletion of mineralocorticoid receptors from macrophages protects against deoxycorticosterone/salt-induced cardiac fibrosis and increased blood pressure. Hypertension. (2009) 54:537–43. 10.1161/HYPERTENSIONAHA.109.131110 19635989

[41] Petry C Fritz G Pfeilschifter J Huwiler A. Inhibition of Rho modulates cytokine-induced prostaglandin E2 formation in renal mesangial cells. Biochim Biophys Acta. (2004) 1636:108–18. 10.1016/j.bbalip.2003.11.007 15164758

[42] Pfaffl MW A new mathematical model for relative quantification in real-time RT-PCR. *Nucleic Acids Res*. (2001) 29:e45 10.1093/nar/29.9.e45 PMC55695 11328886

[43] Sonobe T Schwenke DO Pearson JT Yoshimoto M Fujii Y Umetani K. Imaging of the closed-chest mouse pulmonary circulation using synchrotron radiation microangiography. J Appl Physiol. (2011) 111:75–80. 10.1152/japplphysiol.00205.2011 21527665

[44] Rueden CT Schindelin J Hiner MC Dezonia BE Walter AE Arena ET. ImageJ2: imagej for the next generation of scientific image data. BMC Bioinformatics. (2017) 18:529. 10.1186/s12859-017-1934-z 29187165 PMC5708080

[45] Mourani PM Sontag MK Younoszai A Ivy DD Abman SH. Clinical utility of echocardiography for the diagnosis and management of pulmonary vascular disease in young children with chronic lung disease. Pediatrics. (2008) 121:317–25. 10.1542/peds.2007-1583 18245423 PMC3121163

[46] De Jong S Van Veen TA De Bakker JM Van Rijen HV. Monitoring cardiac fibrosis: a technical challenge. Neth Heart J. (2012) 20:44–8. 10.1007/s12471-011-0226-x 22161127 PMC3247628

[47] Schipke J Brandenberger C Rajces A Manninger M Alogna A Post H. Assessment of cardiac fibrosis: a morphometric method comparison for collagen quantification. J Appl Physiol. (2017) 122:1019–30. 10.1152/japplphysiol.00987.2016 28126909

[48] Martinez-Martinez E Brugnolaro C Ibarrola J Ravassa S Buonafine M Lopez B. CT-1 (Cardiotrophin-1)-Gal-3 (Galectin-3) axis in cardiac fibrosis and inflammation. Hypertension. (2019) 73:602–11. 10.1161/HYPERTENSIONAHA.118.11874 30612490

[49] Dutta S Sengupta P. Men and mice: relating their ages. Life Sci. (2016) 152:244–8. 10.1016/j.lfs.2015.10.025 26596563

[50] Frank JA Pittet JF Wray C Matthay MA. Protection from experimental ventilator-induced acute lung injury by IL-1 receptor blockade. Thorax. (2008) 63:147–53. 10.1136/thx.2007.079608 17901159

[51] Jakkula M Le Cras TD Gebb S Hirth KP Tuder RM Voelkel NF. Inhibition of angiogenesis decreases alveolarization in the developing rat lung. Am J Physiol Lung Cell Mol Physiol. (2000) 279:L600–7. 10.1152/ajplung.2000.279.3.L600 10956636

[52] Humbert M Monti G Brenot F Sitbon O Portier A Grangeot-Keros L. Increased interleukin-1 and interleukin-6 serum concentrations in severe primary pulmonary hypertension. Am J Respir Crit Care Med. (1995) 151:1628–31. 10.1164/ajrccm.151.5.7735624 7735624

[53] Soon E Holmes AM Treacy CM Doughty NJ Southgate L Machado RD. Elevated levels of inflammatory cytokines predict survival in idiopathic and familial pulmonary arterial hypertension. Circulation. (2010) 122:920–7. 10.1161/CIRCULATIONAHA.109.933762 20713898

[54] Cracowski JL Chabot F Labarere J Faure P Degano B Schwebel C. Proinflammatory cytokine levels are linked to death in pulmonary arterial hypertension. Eur Respir J. (2014) 43:915–7. 10.1183/09031936.00151313 24232704

[55] Parpaleix A Amsellem V Houssaini A Abid S Breau M Marcos E. Role of interleukin-1 receptor 1/MyD88 signalling in the development and progression of pulmonary hypertension. Eur Respir J. (2016) 48:470–83. 10.1183/13993003.01448-2015 27418552

[56] Pickworth J Rothman A Iremonger J Casbolt H Hopkinson K Hickey PM. Differential IL-1 signaling induced by BMPR2 deficiency drives pulmonary vascular remodeling. Pulm Circ. (2017) 7:768–76. 10.1177/2045893217729096 28828907 PMC5703124

[57] Voelkel NF Tuder R. Interleukin-1 receptor antagonist inhibits pulmonary hypertension induced by inflammation. Ann N Y Acad Sci. (1994) 725:104–9. 10.1111/j.1749-6632.1994.tb39794.x 8030981

[58] Chada M Nogel S Schmidt AM Ruckel A Bosselmann S Walther J. Anakinra (IL-1R antagonist) lowers pulmonary artery pressure in a neonatal surfactant depleted piglet model. Pediatr Pulmonol. (2008) 43:851–7. 10.1002/ppul.20851 18668691

[59] Trankle CR Canada JM Kadariya D Markley R De Chazal HM Pinson J. IL-1 blockade reduces inflammation in pulmonary arterial hypertension and right ventricular failure: a single-arm, open-label, phase IB/II pilot study. Am J Respir Crit Care Med. (2019) 199:381–4. 10.1164/rccm.201809-1631LE 30418047 PMC6913087

[60] Maniscalco WM Watkins RH D'angio CT Ryan RM. Hyperoxic injury decreases alveolar epithelial cell expression of vascular endothelial growth factor (VEGF) in neonatal rabbit lung. Am J Respir Cell Mol Biol. (1997) 16:557–67. 10.1165/ajrcmb.16.5.9160838 9160838

[61] D'angio CT Maniscalco WM Ryan RM Avissar NE Basavegowda K Sinkin RA. Vascular endothelial growth factor in pulmonary lavage fluid from premature infants: effects of age and postnatal dexamethasone. Biol Neonate. (1999) 76:266–73. 10.1159/000014168 10516393

[62] Lassus P Ristimaki A Ylikorkala O Viinikka L Andersson S. Vascular endothelial growth factor in human preterm lung. Am J Respir Crit Care Med. (1999) 159:1429–33. 10.1164/ajrccm.159.5.9806073 10228106

[63] Maniscalco WM Watkins RH Pryhuber GS Bhatt A Shea C Huyck H. Angiogenic factors and alveolar vasculature: development and alterations by injury in very premature baboons. Am J Physiol Lung Cell Mol Physiol. (2002) 282:L811–23. 10.1152/ajplung.00325.2001 11880308

[64] Bhandari V Choo-Wing R Lee CG Yusuf K Nedrelow JH Ambalavanan N. Developmental regulation of NO-mediated VEGF-induced effects in the lung. Am J Respir Cell Mol Biol. (2008) 39:420–30. 10.1165/rcmb.2007-0024OC 18441284 PMC2551703

[65] Akeson AL Greenberg JM Cameron JE Thompson FY Brooks SK Wiginton D. Temporal and spatial regulation of VEGF-A controls vascular patterning in the embryonic lung. Dev Biol. (2003) 264:443–55. 10.1016/j.ydbio.2003.09.004 14651929

[66] Ben-Av P Crofford LJ Wilder RL Hla T. Induction of vascular endothelial growth factor expression in synovial fibroblasts by prostaglandin E and interleukin-1: a potential mechanism for inflammatory angiogenesis. FEBS Lett. (1995) 372:83–7. 10.1016/0014-5793(95)00956-A 7556649

[67] Hellwig-Burgel T Rutkowski K Metzen E Fandrey J Jelkmann W. Interleukin-1beta and tumor necrosis factor-alpha stimulate DNA binding of hypoxia-inducible factor-1. Blood. (1999) 94:1561–7. 10477681

[68] Kobayashi T Liu X Wen FQ Fang Q Abe S Wang XQ. Smad3 mediates TGF-beta1 induction of VEGF production in lung fibroblasts. Biochem Biophys Res Commun. (2005) 327:393–8. 10.1016/j.bbrc.2004.12.032 15629128

[69] Martin D Galisteo R Gutkind JS. CXCL8/IL8 stimulates vascular endothelial growth factor (VEGF) expression and the autocrine activation of VEGFR2 in endothelial cells by activating NFkappaB through the CBM (Carma3/Bcl10/Malt1) complex. J Biol Chem. (2009) 284:6038–42. 10.1074/jbc.C800207200 19112107 PMC2649103

[70] Ambalavanan N Carlo WA D'angio CT Mcdonald SA Das A Schendel D Cytokines associated with bronchopulmonary dysplasia or death in extremely low birth weight infants. Pediatrics. (2009) 123:1132–41. 10.1542/peds.2008-0526 19336372 PMC2903210

[71] Cayabyab RG Jones CA Kwong KY Hendershott C Lecart C Minoo P. Interleukin-1beta in the bronchoalveolar lavage fluid of premature neonates: a marker for maternal chorioamnionitis and predictor of adverse neonatal outcome. J Matern Fetal Neonatal Med. (2003) 14:205–11. 10.1080/jmf.14.3.205.211 14694976

[72] Mahieu LM De Dooy JJ Ieven MM Bridts CH Stevens WJ. Increased levels of tumor necrosis factor-alpha and decreased levels of interleukin-12 p 70 in tracheal aspirates, within 2 hrs after birth, are associated with mortality among ventilated preterm infants. Pediatr Crit Care Med. (2005) 6:682–9. 10.1097/01.PCC.0000185483.09667.CB 16276336

[73] Bose CL Dammann CE Laughon MM. Bronchopulmonary dysplasia and inflammatory biomarkers in the premature neonate. Arch Dis Child Fetal Neonatal Ed. (2008) 93:F455–61. 10.1136/adc.2007.121327 18676410

[74] Dewberry RM King AR Crossman DC Francis SE. Interleukin-1 receptor antagonist (IL-1ra) modulates endothelial cell proliferation. FEBS Lett. (2008) 582:886–90. 10.1016/j.febslet.2008.02.021 18282478

[75] Jankov RP Luo X Cabacungan J Belcastro R Frndova H Lye SJ. Endothelin-1 and O2-mediated pulmonary hypertension in neonatal rats: a role for products of lipid peroxidation. Pediatr Res. (2000) 48:289–98. 10.1203/00006450-200009000-00005 10960492

[76] Jankov RP Kantores C Belcastro R Yi M Tanswell AK. Endothelin-1 inhibits apoptosis of pulmonary arterial smooth muscle in the neonatal rat. Pediatr Res. (2006) 60:245–51. 10.1203/01.pdr.0000233056.37254.0b 16857764

[77] Helset E Kjaeve J Hauge A. Endothelin-1-induced increases in microvascular permeability in isolated, perfused rat lungs requires leukocytes and plasma. Circ Shock. (1993) 39:15–20. 8481973

[78] Allen SW Chatfield BA Koppenhafer SA Schaffer MS Wolfe RR Abman SH. Circulating immunoreactive endothelin-1 in children with pulmonary hypertension. Association with acute hypoxic pulmonary vasoreactivity. Am Rev Respir Dis. (1993) 148:519–22. 10.1164/ajrccm/148.2.519 8342919

[79] Barnes PJ. Endothelins and pulmonary diseases. J Appl Physiol. (1994) 77:1051–9. 10.1152/jappl.1994.77.3.1051 7836103

[80] Yoshizumi M Kurihara H Morita T Yamashita T Oh-Hashi Y Sugiyama T. Interleukin 1 increases the production of endothelin-1 by cultured endothelial cells. Biochem Biophys Res Commun. (1990) 166:324–9. 10.1016/0006-291X(90)91948-R 2405848

[81] Belik J Jankov RP Pan J Tanswell AK Chronic O2 exposure enhances vascular and airway smooth muscle contraction in the newborn but not adult rat. J Appl Physiol. (2003) 94:2303–12. 10.1152/japplphysiol.00820.2002 12562676

[82] Dumas De La Roque E Smeralda G Quignard JF Freund-Michel V Courtois A Marthan R Altered vasoreactivity in neonatal rats with pulmonary hypertension associated with bronchopulmonary dysplasia: Implication of both eNOS phosphorylation and calcium signaling. PLoS ONE. (2017) 12:e0173044 10.1371/journal.pone.0173044 28235094 PMC5325597

[83] Trittmann JK Velten M Heyob KM Almazroue H Jin Y Nelin LD. Arginase and alpha-smooth muscle actin induction after hyperoxic exposure in a mouse model of bronchopulmonary dysplasia. Clin Exp Pharmacol Physiol. (2018) 45:556–62. 10.1111/1440-1681.12909 29266319 PMC5991998

[84] Frangogiannis NG. Interleukin-1 in cardiac injury, repair, and remodeling: pathophysiologic and translational concepts. Discoveries. (2015) 3:e41. 10.15190/d.2015.33 26273700 PMC4532433

[85] De Boer RA Voors AA Muntendam P Van Gilst WH Van Veldhuisen DJ Galectin-3: a novel mediator of heart failure development and progression. Eur J Heart Fail. (2009) 11:811–7. 10.1093/eurjhf/hfp097 19648160

[86] Dobaczewski M Frangogiannis NG. Chemokines and cardiac fibrosis. Front Biosci. (2009) 1:391–405. 10.2741/s33 19482709 PMC2798729

[87] Suthahar N Meijers WC Sillje HHW De Boer RA. From inflammation to fibrosis-molecular and cellular mechanisms of myocardial tissue remodelling and perspectives on differential treatment opportunities. Curr Heart Fail Rep. (2017) 14:235–50. 10.1007/s11897-017-0343-y 28707261 PMC5527069

[88] Harding P Carretero OA Lapointe MC. Effects of interleukin-1 beta and nitric oxide on cardiac myocytes. Hypertension. (1995) 25:421–30. 10.1161/01.HYP.25.3.421 7875768

[89] Ogawa T De Bold AJ. Brain natriuretic Peptide production and secretion in inflammation. J Transplant. (2012) 2012:962347. 10.1155/2012/962347 23251786 PMC3515950

[90] Bui CB Pang MA Sehgal A Theda C Lao JC Berger PJ. Pulmonary hypertension associated with bronchopulmonary dysplasia in preterm infants. J Reprod Immunol. (2017) 124:21–9. 10.1016/j.jri.2017.09.013 29035757

