# Supplementary material for: Interleukin-1 Receptor Antagonist Protects Newborn Mice Against Pulmonary Hypertension
Source: Front Immunol. 2019 Jul 11;10:1480. doi: 10.3389/fimmu.2019.01480 (PMC6637286; doi:10.3389/fimmu.2019.01480)
Supplement: Supplementary file 1 [file Image_1.pdf]

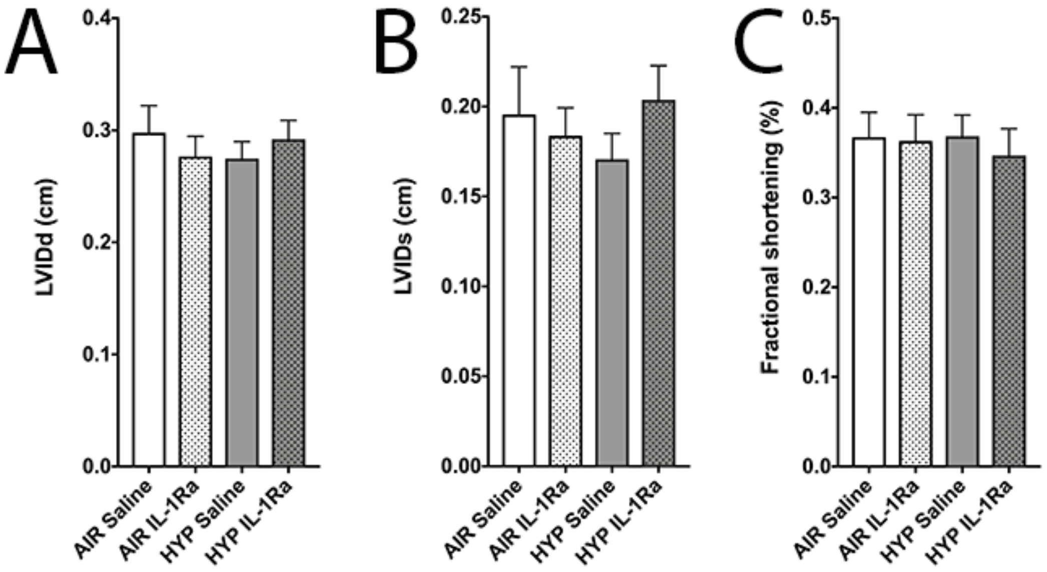

Supplementary Figure 1.

Echocardiography was performed on 28d mice after exposure to LPS and 65% O_2 to assess LV function. Measurements included left ventricular internal diameter at end-diastole (LVIDd; A) and end-systole (LVIDs; B) and fractional shortening (C).
