# Supplementary material for: Interleukin-1 Receptor Antagonist Protects Newborn Mice Against Pulmonary Hypertension
Source: Front Immunol. 2019 Jul 11;10:1480. doi: 10.3389/fimmu.2019.01480 (PMC6637286; doi:10.3389/fimmu.2019.01480)
Supplement: Supplementary file 2 [file Image_2.pdf]

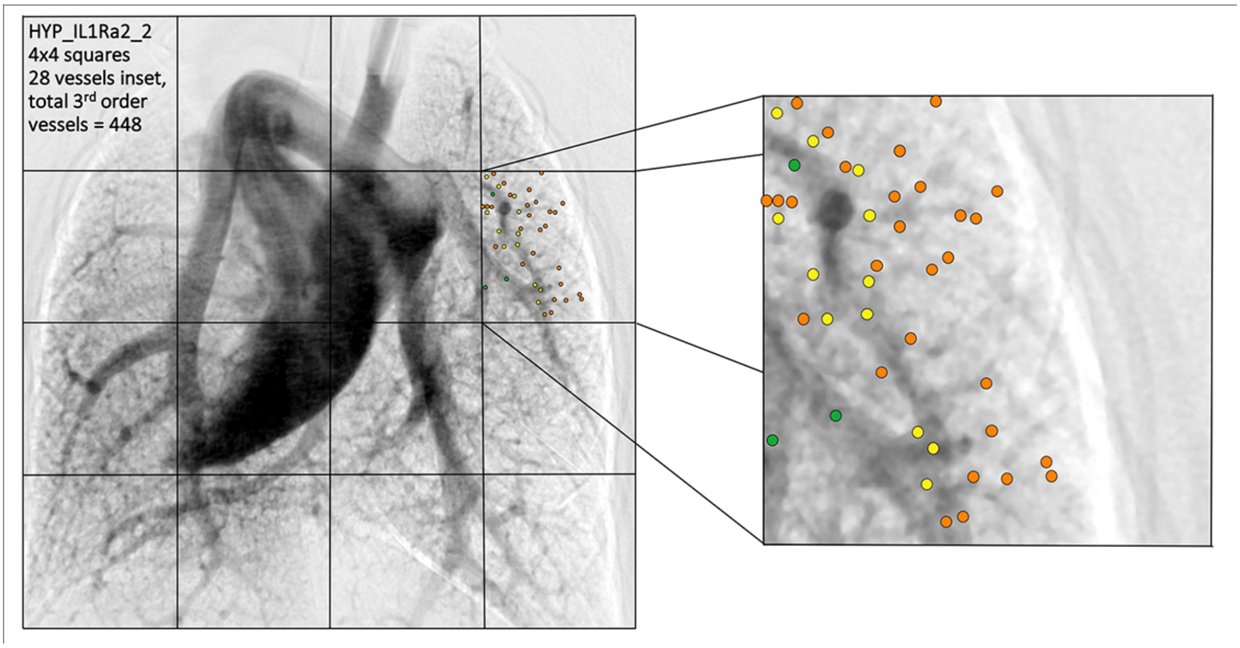

Supplementary Figure 2.

Cine-angiography was performed on 60d mice treated with IL-1Ra for the first 28d of life. Each vessel was manually marked with a color-coded dot (green-1st, yellow-2nd and orange 3rd) and all dots were counted automatically with ImageJ software (Schneider et al., 2012). Representative hyperoxia IL-1Ra-treated mouse shows estimated number of 3rd generation branches in middle right lobe and all lobes (dots not shown for clarity).
