# Supplementary material for: Interleukin-1 Receptor Antagonist Protects Newborn Mice Against Pulmonary Hypertension
Source: Front Immunol. 2019 Jul 11;10:1480. doi: 10.3389/fimmu.2019.01480 (PMC6637286; doi:10.3389/fimmu.2019.01480)
Supplement: Supplementary file 3 [file Table_1.docx]

**Supplementary Table 1. List of RT-PCR primers**

| **Gene Name** | **Gene Symbol** | **Assay ID** |
| --- | --- | --- |
| Galectin-3 | *Lgals3* | Mm00802901_m1 |
| Chemokine (C-C motif) ligand 2 | *Ccl2* | Mm00441242_m1 |
| Natriuretic peptide type B | *Nppb* | Mm01255770_g1 |
| Beta actin | *Actb* | Mm00607939_s1 |
